# ‘Far Away from Home’: adolescent inpatient admissions far from home, out of area or to adult wards: a national surveillance study

**DOI:** 10.1136/bmjment-2023-300843

**Published:** 2023-12-09

**Authors:** Josephine Holland, James Roe, Boliang Guo, Morenike Dasilva-Ellimah, Anne-Marie Burn, Bernadka Dubicka, Tamsin Ford, Adam P Wagner, Saeed Nazir, Anthony James, Richard Morriss, Kapil Sayal

**Affiliations:** 1 Mental Health and Clinical Neurosciences, Institute of Mental Health, University of Nottingham, Nottingham, UK; 2 National Institute for Health and Care Research Applied Research Collaboration (ARC) East Midlands, University of Nottingham, Nottingham, UK; 3 National Institute for Health and Care Research Applied Research Collaboration (ARC) East of England, University of Cambridge, Cambridge, UK; 4 Department of Child and Adolescent Psychiatry, University of York, York, UK; 5 Department of Psychiatry, University of Cambridge, Cambridge, UK; 6 National Institute for Health and Care Research Applied Research Collaboration (ARC) East of England, University of East Anglia, Norwich, UK; 7 Nottinghamshire Healthcare NHS Foundation Trust, Nottingham, UK; 8 Oxford Health NHS Foundation Trust, Oxford, UK; 9 Royal College of Psychiatrists, London, UK

**Keywords:** PSYCHIATRY, Child & adolescent psychiatry

## Abstract

**Background:**

The increasing prevalence and acuity of mental disorders among children and adolescents have placed pressure on services, including inpatient care, and resulted in young people being admitted at-distance or to adult wards. Little empirical research has investigated such admissions.

**Objective:**

To determine the incidence, clinical characteristics and 6-month outcomes of patients aged 13–17 years old admitted at-distance (>50 miles from home or out of region) to general adolescent psychiatric wards or to adult psychiatric wards.

**Methods:**

Surveillance over 13 months (February 2021–February 2022) using the Child and Adolescent Psychiatry Surveillance System including baseline and 6-month follow-up questionnaires.

**Findings:**

Data were collected about 290 admissions (follow-up rate 99% (288 of 290); sample were 73% female, mean age 15.8 years). The estimated adjusted yearly incidence of at-distance admission was 13.7–16.9 per 100 000 young people 13–17 years old. 38% were admitted >100 miles from home and 8% >200 miles. The most common diagnoses at referral were depression (34%) and autism spectrum disorder (20%); other common referral concerns included suicide risk (80%), emotional dysregulation (53%) and psychotic symptoms (22%). Over two-fifths (41%) waited ≥1 week for a bed, with 55% waiting in general hospital settings. At 6-month follow-up, 20% were still in hospital, the majority in at-distance placements.

**Conclusions:**

At-distance and adult ward admissions for patients aged <18 remain an ongoing challenge for healthcare provision and have an impact on acute hospital resource use.

**Clinical implications:**

Long waits in non-specialist settings increase pressure across the healthcare system, highlighting the need to improve local service provision and commissioning to reflect identified clinical needs.

**WHAT IS ALREADY KNOWN ON THIS TOPIC**

- At-distance and adult ward admissions in Child and Adolescent Mental Health Services (CAMHS) are often labelled as ‘inappropriate’, and national policies describe intentions to eliminate these types of admissions in the future but there has been little research into the impacts of these admissions.

**WHAT THIS STUDY ADDS**

- Young people for whom admissions are requested on an emergency basis frequently wait a week or more for a CAMHS bed, with most waiting in an acute hospital setting.
- Clinical risk is a main driver for these admissions, with suicide risk present in 80% and risk management requested in almost every case.
- The average length of at-distance admissions is 15 days longer than the overall average length of inpatient CAMHS admissions, with a delay to discharge in one-third of cases.

**HOW THIS STUDY MIGHT AFFECT RESEARCH, PRACTICE OR POLICY**

- Commissioning and local service provision driven by identified needs, as well as close working between inpatient and community teams, other specialties, social care and education, is required to reduce the need for at-distance and adult ward admissions.

## Background

Rates of mental disorder among young people are increasing.^1 2^ This is reflected in increases of referrals to Child and Adolescent Mental Health Services (CAMHS),^3^ mental health crisis presentations to the emergency department (ED),^4^ admissions to paediatric wards^5^ and compulsory hospital admissions (eg, through the use of the Mental Health Act (MHA) in England),^6^ suggesting that the acuity of presentations is also increasing. Where a young person’s mental health needs and risks have reached a level of severity and complexity that care cannot be safely provided in the community, an inpatient psychiatric admission (to a general adolescent unit, if aged 13–17 years) might be sought. If a bed in a local unit cannot be found in a timely manner, this might result in an admission to an at-distance unit. These types of admissions are a frequently discussed and controversial topic in the media and in policy statements,^7 8^ with concerns about time spent away from family, friends, school and local support networks during this formative phase of life. The admission of patients under-18 to adult psychiatric wards has also been a cause of concern.^9 10^ Despite this, little empirical research has systematically investigated the scale, impacts and outcomes of these types of admissions in the UK or elsewhere. Surveillance studies, when compared with routinely collected administrative data, allow for the collection of detailed information such as clinical characteristics, reasons for admission and diagnosis. They also enable the collection of follow-up data about clinical and service use outcomes.

## Objective

To systematically gather clinical information about at-distance (far from home or out of region) and adult ward admissions in England over a 13-month period to investigate their incidence, clinical characteristics and 6-month outcomes.

## Methods

### Study design

A national surveillance study in England to determine the surveillance incidence, clinical characteristics and outcomes of patients 13–17 years old who had been admitted to a: (1) general adolescent unit (GAU; that is, a general psychiatric ward for those under-18s) at distance from home or (2) adult psychiatric ward, during a 13-month surveillance period (1 February 2021–28 February 2022) as reported by consultant child and adolescent psychiatrists (‘at distance’ was defined as over 50 miles from their home address or outside their National Health Service (NHS) commissioning region area (in England, this reflected 10 NHS regions or ‘commissioning areas’) as illustrated in online supplemental appendix 1). Case details were collected using two methods outlined below.

10.1136/bmjment-2023-300843.supp1 Supplementary data

Surveillance data were triangulated with recorded admission numbers during the surveillance period from NHS England (the lead and central administrative organisation).

### Funding statement

This study is funded by the National Institute for Health and Care Research (NIHR) Applied Research Collaboration East Midlands. The views expressed are those of the authors and not necessarily those of the NIHR or the Department of Health and Social Care. The study researchers acted independently of the funder. All authors had full access to all of the data (including statistical reports and tables) in the study and can take responsibility for the integrity of the data and the accuracy of the data analysis.

### Case notification

#### Strand 1: Child and Adolescent Psychiatry Surveillance System

Child and Adolescent Psychiatry Surveillance System (CAPSS) is a well-established method of active surveillance to support the epidemiological study of rare mental health disorders or events among children and adolescents in the UK (see previous CAPSS studies, for example, Catch-Us^11^). Hosted by the Royal College of Psychiatrists, consultant child and adolescent psychiatrists are encouraged to register on the CAPSS database and, each month, receive an email e-card which asks them to indicate whether they have seen an eligible case for up to two surveillance studies. For this study (called *‘*Far Away from Home*’*), consultants were asked if they had seen a case meeting any of the criteria below:

A patient 13–17 years old admitted to:

- Any adult psychiatric ward.
- A GAU over 50 miles from their home (defined by shortest road journey).
- A GAU outside their NHS commissioning region area.

Participants were asked to exclude admissions to specialised psychiatric units such as: eating disorder units; learning disability/autism units; forensic/secure units or psychiatric intensive care units (PICUs).

Each month, the CAPSS administrator notified study researchers of the email addresses of consultants who had reported a case and the number of cases they had reported. Reporting consultants were then contacted by the research team with a request to complete a baseline questionnaire about each case (with four ways to respond: via secure electronic link to an online REDCap questionnaire, secure email with a fillable PDF document, postal questionnaire or telephone call with a researcher). Reporting consultants were initially sent email reminders to complete the questionnaire, followed by telephone contact. For eligible cases, 6 months after the date of admission, the consultant was asked to complete a follow-up questionnaire.

#### Strand 2: additional direct case reporting

Some consultants who had participated in strand 1 contacted the study team directly to report an eligible case or completed the baseline questionnaire online. For example, consultants who held a lead role within a regional provider collaborative (during the course of the study, NHS policy changes were implemented to enable closer regional oversight of admissions through the establishment of 18 provider collaboratives) were often able to use their knowledge about admissions and the data recorded by their provider collaborative to identify eligible cases.

**Figure 1:**
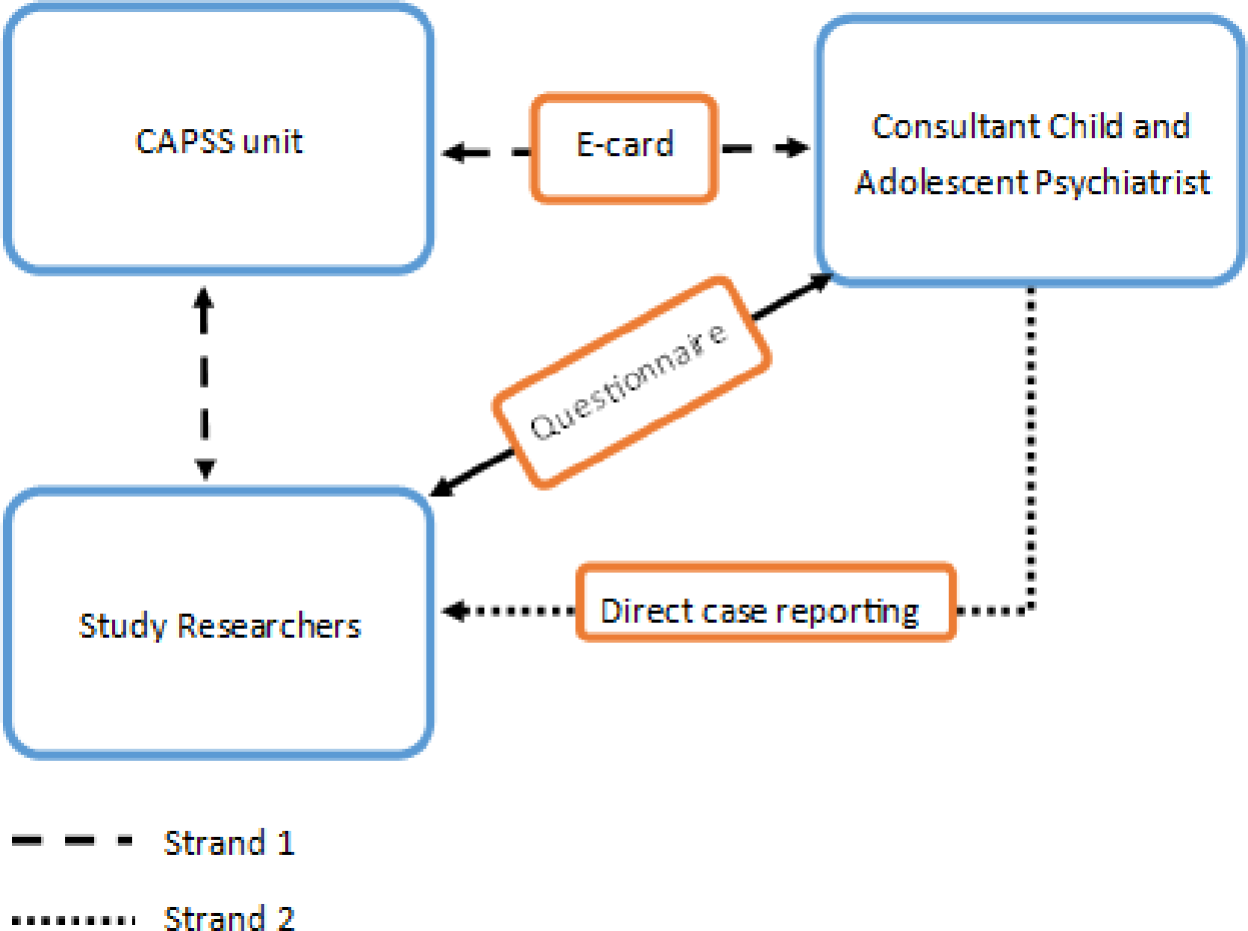
The two strands of case reporting used in the study. Consultants were able to report cases prompted by the Child and Adolescent Psychiatry Surveillance System (CAPSS) or directly to the research team.

Figure 1 shows the two strands via which consultants reported cases to the study.

### Study questionnaires

#### Baseline questionnaire

This was designed to be completed from information CAMHS clinicians provide on the referral form requesting an inpatient admission (NHS England Form 1). The questionnaire asked: patient’s home postcode (for determining the distance between home and the admitting unit, and home NHS region); NHS number; date of admission; and demographic details including date of birth (to calculate age), sex, whether non-binary gender, and ethnicity. The questionnaire enquired about the reasons for requesting admission (including clinical diagnosis and presence of risks), whether the young person was known to services, why an at-distance or adult ward admission occurred and whether it was a planned or emergency admission, which treatments/interventions were requested from the admission, whether the admission was voluntary or compulsory (under the MHA), and where the young person had waited for their admission and for how long.

#### Follow-up questionnaire

The 6-month (from admission date) follow-up asked about the status of the young person and whether they transferred to another unit during their admission, which interventions/treatments were started as an inpatient, involvement of the community team during admission and, if applicable, date of and diagnosis at discharge and whether discharge had been delayed for any reason.

Both questionnaires also had optional free-text boxes for respondents to provide additional detail.

### Determining eligibility

To establish whether a reported case met the study criteria of being admitted more than 50 miles away from their home, distances between associated postcodes were calculated using open access online software (https://www.freemaptools.com/distance-between-uk-postcodes.htm). ‘Free Map Tools’ uses geographical information systems technology to determine the shortest route along a road network and has been used in previous studies.^12^

To establish whether a case was admitted outside the local NHS commissioning region, the region of the home address and the admitting unit were determined and compared. The provider collaborative region was also similarly derived to explore the impact of this policy change.

### Analysis

To calculate incidence, the observed sample size was adjusted to a 12-month estimate and compared with March 2021 Office of National Statistics census estimates of young people 13–17 years old. Adjusted rates based on the methodology used by Janssens *et al*^11^ were calculated and two assumptions were made. Assumption 1 was that the observed incidence rate also applied to half of the non-returned e-cards because consultants with cases to report might be more likely to respond. Assumption 2 was that the observed incidence applied to all non-returned e-cards (ie, no difference in incidence between cases that were and were not reported).

For the quantitative data, normally distributed data were summarised with mean, SD, n, minimum and maximum. Skewed data were summarised by median, IQR, n, minimum and maximum. Categorical data were summarised using frequency (percentage). All analyses were completed using STATA V.17.^13^

The qualitative data provided through free-text responses were analysed using conventional content analysis as described by Hsieh and Shannon.^14^ This involved familiarisation with the data, initial coding, grouping of codes into categories and then combination of categories until main categories emerged.

## Findings

### Cases

**Figure 2:**
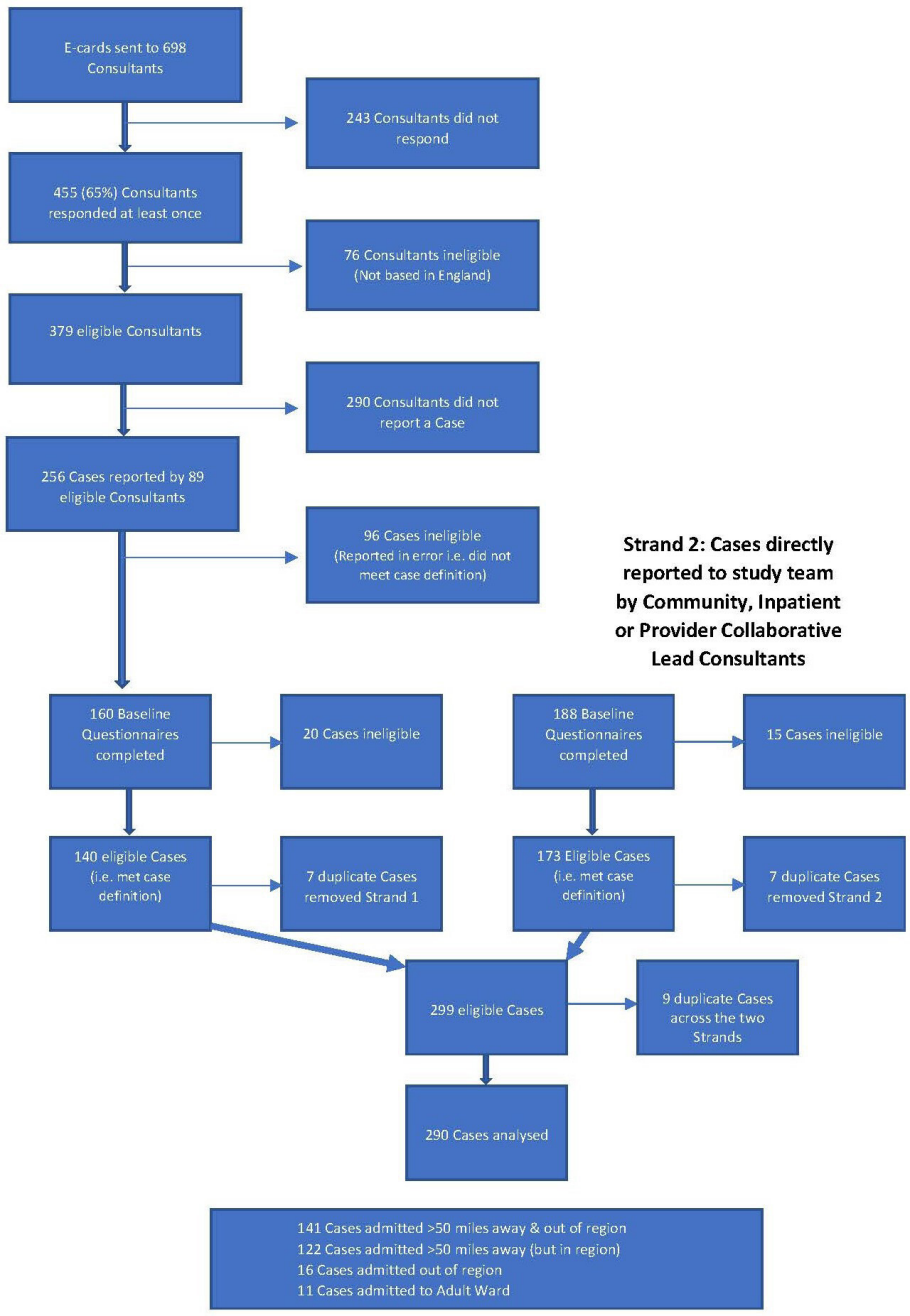
A flow diagram of how the sample of 290 eligible cases was derived across the two reporting strands.

Through CAPSS, 698 consultant child and adolescent psychiatrists were sent monthly e-cards. Sixty-five per cent of consultants responded to at least one e-card during the study period, with a mean monthly response rate of 46%. Figure 2 presents the flow diagram to outline how the sample of 290 eligible cases was derived across the two reporting strands. Patient NHS numbers and admission dates were used to determine duplicate cases both within and between the strands. Where there was duplication, the information was reviewed and combined to ensure a single complete record for the admission. Completion rate of 6-month follow-up questionnaires was very high, with data obtained for 288 (99%) cases.

**Figure 3:**
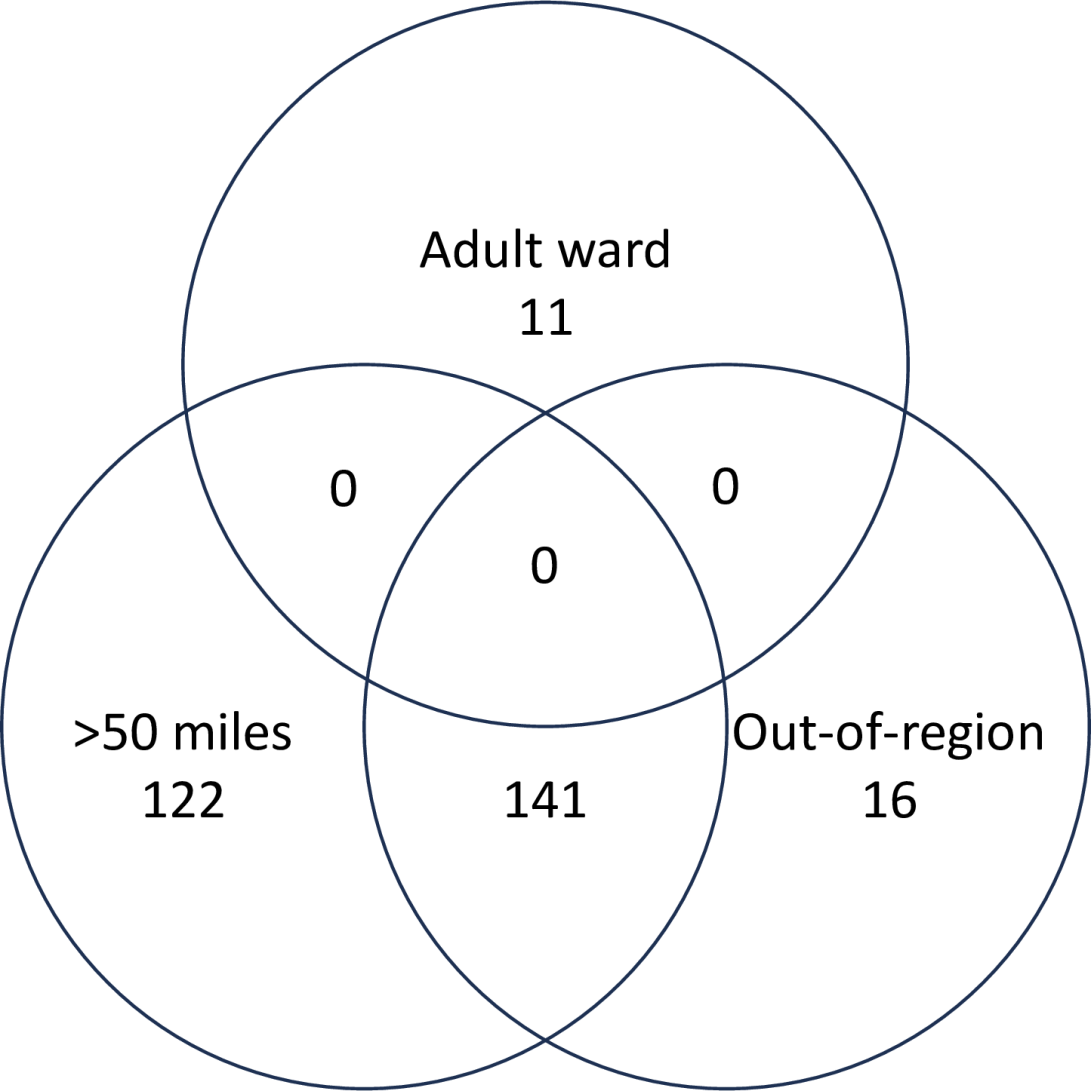
Overlap of eligible cases (n=290) by type of admission. During the course of the study, policy changes were implemented to enable regional oversight of admissions through the establishment of 18 lead provider collaboratives. When the sample was recategorised according to provider collaboratives, there were a few differences due to the differences in geographical boundaries and the ability of provider collaboratives to use units outside of their National Health Service region. For example, whereas 157 young people were admitted out of region, 162 were admitted outside their provider collaborative. These findings suggest that during the period of study surveillance, the introduction of provider collaboratives did not appear to reduce the number of admissions out of area; however, it should be noted that many provider collaboratives were not fully established during the surveillance period (February 2021–February 2022).

As shown in Figure 3, all adult ward admissions were within region and <50 miles from home.

### Incidence of admissions

Table 1 shows incidence calculations, including adjusted rates, for at-distance admissions. During the surveillance period, administrative data from NHS England recorded 2025 admissions to general adolescent units, of which 534 were at distance (personal communication). Our study captured detailed information about 279 (52.3%) of these admissions.

**Table 1:** Calculation of the estimated rate of at-distance and adult ward admissions (per 100 000 people aged 13–17 years per annum)

	At-distance admission	Adult ward admission
Observed incidence for people 13–17 years old (95% exact Poisson CI)		
Observed cases	279	11
Estimated 12-month rate based on census data	7.8 (6.9, 8.9)	Not calculated due to low sample size
Correction for non-returned notification cards		
Returned*	46.3%	
No response*	53.8%	
Assumption 1 (the same incidence applied to half non-returned)	(26.9+53.8)/46.3=coefficient 1.7	Not calculated due to low sample size
Assumption 2 (the same incidence applied to all non-returned)	100/46.3=coefficient 2.2	Not calculated due to low sample size
Combined coefficients		
Adjusted incidence rate 1=incidence rate×correction for unreturned notification cards (assumption 1)	7.8×1.7=13.7	Not calculated due to low sample size
Adjusted incidence rate 2=incidence rate×correction for unreturned notification cards (assumption 2)	7.8×2.2=16.9	Not calculated due to low sample size
Administrative data from NHS England (95% exact Poisson CI)		
Recorded number of admissions	534	259†
Recorded incidence rate	14.8 (13.6, 16.2)	7.3 (6.5, 8.3)

*The mean monthly response rate to e-cards was 46.33%.

†NHS England recorded 259 admissions of under-18s to adult wards; however, they were unable to specify how many of these were young people referred for admission to a GAU. It is likely that some of these young people were referred for more highly specialist inpatient care.

GAU, general adolescent unit; NHS, National Health Service.

### Demographics

The demographics of the young people in our sample are shown in Table 2. Among the young people admitted at-distance within this sample (n=279), 11.8% of males were of black ethnicity; the comparable proportion from the NHS England triangulation data (which record young people’s gender rather than sex) was 9.6%. Of the young people (n=11) admitted to adult wards, 72.7% were female and their mean age was 16.8 years.

**Table 2:** Sample demographic characteristics

Characteristics	Male sex n (%) (N=79)	Binomial exact 95% CI	Female sex n (%) (N=211)	Binomial exact 95% CI	Total n (%) (N=290)	Binomial exact 95% CI
Non-binary gender	3 (3.8)		16 (7.6)		19 (6.6)	
Age						
16–17 years	60 (76.0)		123 (58.3)		183 (63.1)	
Ethnicity						
White*	54 (68.4)	56.9, 78.4	166 (78.7)	72.5, 81.0	220 (75.9)	70.5, 80.7
Asian*	5 (6.3)	2.1, 14.2	7 (3.3)	1.3, 6.7	12 (4.1)	2.2, 7.1
Black*	10 (12.7)	6.2, 22.1	3 (1.4)	0.2, 4.1	13 (4.5)	2.4, 7.5
Mixed or other*	4 (5.1)	1.4, 12.5	20 (9.5)	5.9, 14.3	24 (8.3)	5.4, 12.1
Not known	6 (7.6)	2.8, 15.8	15 (7.1)	4.0, 11.5	21 (7.2)	4.5, 10.9

*In the original questionnaire, the consultant had the opportunity to select: white, white and black Caribbean, white and black African, white and Asian, any other mixed background, Indian, Pakistani, any other Asian background, Caribbean, African, any other (please write) or ethnicity not known. However, to protect the anonymity of young people, ethnicities have been combined to avoid having cell sizes of two or less.

### Clinical characteristics

Prior to their admission, 84.1% of the sample were known to their local CAMHS; of these, 63.5% had been known for over 6 months.

Of these admissions, 235 (81.0%) were requested as emergencies, including all the adult ward admissions, with over half (52.8%) involving the MHA. Common reasons for requesting admission included suicide risk (80.0%), emotional dysregulation (53.5%) and psychotic symptoms (22.4%). For 19.0% of the admissions, the young person had no mental health disorder diagnosis at referral, 38.3% had one diagnosis and 42.8% had two or more diagnoses. Table 3 shows the most common diagnoses at referral and discharge.

**Table 3:** The most common diagnoses at referral and at discharge

Diagnosis	At referral n (%) (n=290)	Discharge/6-month follow-up n (%) (n=288)
Depression	100 (34.5)	101 (35.1)
Autism spectrum disorder	59 (20.3)	64 (22.2)
Psychosis	45 (15.5)	39 (13.5)
Eating disorder	39 (13.5)	30 (10.4)
Anxiety disorder	38 (13.1)	39 (13.5)
Emerging personality disorder/personality disorder	22 (7.6)	33 (11.5)
Post-traumatic stress disorder	20 (6.9)	31 (10.8)

All diagnoses present in >5% of the sample at both referral and discharge.

### Reasons for at-distance or adult ward admission

The most common reasons for these admissions included a lack of local CAMHS beds (73.5% of cases) or local unit-related reasons (eg, shortage of staff, patient mix) despite bed availability (22.1%). Content analysis where respondents provided free-text information revealed three main categories.

#### Category 1: no local bed available

Lack of local CAMHS bed availability was mentioned in 60 comments. Of these, seven reported the local unit had closed, mainly due to temporary issues such as maintenance work or staffing issues but also, in one example, by the Care Quality Commission. Also, 14 comments mentioned that although the bed was within region, it was still over 50 miles away.

##### Subcategory: no local bed with the specialist skill required

Seven comments reported a lack of local beds with required expertise, which mostly related to the management of eating difficulties.

Professionals - unit that can nasogastric feed, under restraint if needed. Unit with experience of eating disorders and disordered (non-anorexic) eating.

Proximity to other services was also important—two comments reported the need to be near a PICU.

##### Subcategory: young person/carer attitudes towards at-distance admission

Seven comments reported that young people and/or parents/carers would have preferred a local admission. One psychiatrist reported that a young person chose to be admitted to a local adult bed to avoid an at-distance admission.

They were keen to be local - thus admitted to a local adult bed as no adolescent bed was available.

#### Category 2: young person/parent objection to local unit available

In instances where a local unit had been an option, six comments reported that an at-distance unit was chosen due to the young person’s and/or parent’s objection to the local unit—five reported a negative experience at the local unit and one reported knowing other young people there.

Patient associates the nearest GAU with a traumatic memory of witnessing a peer’s tragic death on the ward. Alternative hospitals in the area did not have a bed available.

There were also two reports of young people or their parents requesting a particular unit. One was due to the young person’s familiarity with the unit’s staff and the other because the young person’s parents were temporarily living elsewhere.

Transferred from a unit closer to home as the patient’s family were temporarily staying locally so this unit was closer for them to visit than their home address.

#### Category 3: exceptionally acute need for a bed

A total of 14 comments mentioned a young person being admitted to the first available bed due to the acuity of their presentation. Consultants referred to young people deteriorating in the community (n=6), deteriorating on a paediatric ward (n=1) or requiring intense input (n=2) or crisis management (n=5). One consultant reported that a young person was initially due to be admitted locally but was sent to an at-distance unit instead as the urgency increased:

They had no choice over the matter, given the acuity of presentation.

Escalating challenging behaviours on a paediatric ward, difficult to contain, police called around five times to the ward, needed quick admission.

### Location and length of wait for a bed

Over one-fifth (23.5%) of the young people waited over 10 days for a bed (longest waiting time consultants could select in the questionnaire). A further 17.6% of the young people waited for 7–10 days. Nine per cent of young people experienced a wait of less than a day for a bed.

The majority of these young people waited in general hospital settings (40.0% paediatric ward, 7.9% adult medical ward and 7.2% ED). One in 10 (10.7%) had to wait in Section 136 suites, a specialist holding suite usually located at an adult psychiatric hospital, designed to hold people for no longer than 24 hours while awaiting a MHA assessment.

### Nature and course of admission

#### Distance

Among those admitted to GAUs (n=279), 54.5% were between 50 and 100 miles from home, 22.2% 100–150 miles, 9.7% 150–200 miles and 7.9% more than 200 miles. All the adult ward admissions were within area and <50 miles from home.

#### Length of stay and care received

The median length of stay was 75 days (IQR 34–142 days) for the whole sample, 36 days for those admitted to adult wards and 76 for those admitted at-distance.

At the point of referral, the most common types of care requested from the admission were risk reduction/management (97.6%) and assessment/monitoring (95.5%); less commonly requested were psychological therapy (65.5%), occupational therapy (45.5%) and family therapy (40.0%). When received care was recorded at follow-up, 99.0% had received assessment/monitoring, 96.9% risk reduction/management, 77.5% medication initiated (with 85.4% receiving reviews of medication), 76.3% psychological therapy, 64.5% occupational therapy and 52.3% family therapy.

#### Transfers

Just under one-fifth (19.0%) of young people were known to have transferred to another ward during their admission (36.4% of those admitted to adult wards and 18.3% of those admitted at distance). Among the sample, 23 (7.9%) were transferred to a GAU within their commissioning region (known as repatriated). For those who were transferred (n=55), the median duration of the stay on their initial ward was 38.5 days. Of those who had transferred unit, 27.3% went to another GAU more than 50 miles from their home (ie, they experienced two at-distance placements during the course of the same admission), 5.5% went to an adult psychiatric ward, 7.3% went to a low secure unit and 16.4% to a PICU.

### Discharge and delays to discharge

For 93.4% of the sample, clinicians from their local CAMHS team attended at least one ward meeting while they were an inpatient, mainly through video call (92.2%). For 87.5%, their local CAMHS team attended a discharge meeting, again most commonly through video call (94.4%).

At 6 months after the admission date given, 59 (20.3%) of the young people were still in hospital; less than 5* young people were still on an adult ward (*exact number withheld to protect anonymity), but 71.2% of the young people in hospital at 6 months were still placed in a hospital which was at distance.

Discharge was reported as being delayed for 34.1% of the sample. The most common reasons were distance from home limiting in-reach from the local CAMHS team (18.5%), and distance from home causing difficulty organising local social care support (17.1%). When asked to list ‘other’ reasons, 20 ‘other reasons’ were noted, with two main categories that reflected *‘*social circumstances’ and ‘patient factors’.

#### Category 1: social circumstances

This occurred in 13 comments, 5 mentioning that the young person did not have a suitable discharge destination. One comment reported that the young person’s care home refused to take the young person back.

Four comments related to issues with the young person’s family, such as breakdown in the familial relationship and parental anxiety surrounding discharge. In addition, a couple of comments reported that the young person was waiting for a suitable provision to be able to admit them.

No accommodation. Care home handed in their notice at discharge.

… complex family issues complicating pursuing alternative accommodation.

#### Category 2: patient factors

Four comments mentioned that the discharge was delayed due to patient factors, all related to complexity or deterioration in the young person’s clinical condition.

Delayed discharge due to needing more intensive community package of care.

## Discussion

The findings from this unique national surveillance study show that most at-distance and adult mental health ward admissions for people 13–17 years old are emergency admissions for young people who have been detained under the MHA. Sample characteristics highlighted a predominance of female and older adolescents, with many having comorbid mental health diagnoses and high risks, most notably suicide risk (80% of cases). The salience of risk associated with these presentations is highlighted by risk reduction being the most frequently requested intervention at admission, requested almost universally.

The most commonly reported diagnosis at admission was depression, suggesting that treatment-resistant or complex depression is an important driver for requesting inpatient care. Over one-fifth of young people captured in our study at baseline and at follow-up had a diagnosis of autism spectrum disorder (ASD). Specific policies such as Care, Education and Treatment reviews were developed to help young people with ASD avoid hospital admission wherever possible and minimise length of stay^15 16^; however, as shown by these findings, young people with autism are still requiring admission and staying for an extended period. Our study however did not enquire whether these initiatives had been implemented for these young people, and this will be important to capture in future work.

Our findings of a greater proportion of female young people and older adolescents in this sample are consistent with recent community-based studies investigating the prevalence of mental disorders in the wider population.^17^ In our study, however, there was an over-representation of males of black ethnicity compared with the general population, which is concerning. This finding is in keeping with government reports showing an over-representation of black young people detained under the MHA.^18^ However, since our study has a relatively small sample size, this finding should be interpreted with caution. In addition, as our study did not collect data about local admissions, it is not possible to conclude whether this represents a bias towards males of black ethnicity being more likely to be admitted generally, or specifically at distance. Routine ethnic minority monitoring of admissions by NHS England may help to further investigate this possibility.

In mental health access standards, there is an expectation that those requiring urgent or emergency care should receive this within 24 hours.^19^ However, despite 80% of these admissions being requested as an emergency, our study found that over 40% of young people waited for a week or more for a bed, with many waiting in general hospital settings, particularly paediatric wards. These findings fit with surveys of paediatric doctors in the UK who reported that mental health admissions to paediatric wards make up more than a quarter of their admissions.^5^ Such points of ‘cross-over’ can create tension between staff working in general hospital and mental health settings—collaborative working across services and agencies is likely to be fruitful and may prevent risk escalation during this period. Future commissioning of paediatric and CAMHS beds should take this into account.

The median length of stay of the admissions in this study was 75 days; for those admitted at distance, this was 76 days, which was 2 weeks longer than previous figures of the average length of CAMHS admission of 61 days.^20^ Over one-fifth of the young people in our study were still in hospital after 6 months. This is a very long time in a person’s life, away from friends, family, school and usual daily activities at a seminal stage of development. The ‘Getting It Right First Time’ report^21^ found that length of stay in CAMHS was less than 60 days in only 39% of cases. Since our study focused on at-distance admissions, this suggests that many at-distance admissions are longer than local admissions, which was further corroborated by responding consultants who reported that discharge was delayed in one-third of cases. This is very likely to result in higher NHS costs and may result in greater costs more widely if reintegration is more difficult for the young person (eg, being away from school for such a long time).

Our study has several strengths. The use of a national surveillance system ensured that cases were identified across the country and that the sample was representative across England. The triangulation with administrative data from NHS England showed that our study collected detailed clinical and contextual data on over half of the at-distance admissions during the surveillance period. The 99% follow-up completion rate ensured comprehensive capture of 6-month clinical outcome data for these young people, which is not easily possible with administrative data. However, limitations of our study included modest monthly response rates (46%). Some responding consultants explained that their unit received so many at-distance admissions that they did not have time to complete questionnaires about each case; therefore, our understanding of the admissions to these units was not as comprehensive. The low reporting of adult ward admissions was likely affected by the fact that general adult psychiatrists who looked after the young people on the wards would not have been within the CAPSS database. Another limitation was the voluntary nature of participation among consultants, with the possibility that consultants with strong opinions about these types of admissions, positive or negative, were perhaps more likely to respond.

## Clinical implications

As shown by the findings of this investigation, at-distance and adult ward admissions continue to affect many people under-18s requiring psychiatric inpatient admission. Provider collaboratives within each region, once fully established, aim to change this, allowing more regional clinical oversight of admissions; however, more time is needed to assess the impact of this change.

These admissions are mainly emergencies and driven by risks which cannot currently be safely managed in community settings. The majority of this sample was known to CAMHS prior to referral, usually for over 6 months, suggesting that more needs to be done to identify and manage escalating risk at earlier stages to prevent it reaching the level for requiring admission.

The high proportion of young people with ASD in this sample is concerning. Previous studies have shown that young people with ASD can present to EDs with higher acuity than those without ASD, particularly with acute behavioural disturbance.^22^ Despite the implementation of measures to reduce admissions in this group, it seems current care provision is not meeting their needs, with many additional barriers to mental health support for these young people.^23^

Young people are spending significant amounts of time waiting for an available psychiatric bed even after the decision to admit has been made. Acute hospital beds are often having to provide a safe ‘holding’ place in the interim. However, since the aim is for all CAMHS admissions to be as short as possible, such interim placements could be used more effectively by commencing assessment and treatment processes within them. To implement this, intensive involvement of CAMHS staff with the young person in the general hospital is needed, alongside support and training for those who work in the place where the young person is waiting. To achieve this, more joint service commissioning and planning across CAMHS and paediatric services are essential.

## Data Availability

Data are available upon reasonable request.
