# Supplementary figures and images for: ‘Far Away from Home’: adolescent inpatient admissions far from home, out of area or to adult wards: a national surveillance study
Source: BMJ Ment Health. 2023 Dec 9;26(1):e300843. doi: 10.1136/bmjment-2023-300843 (PMC10728958; doi:10.1136/bmjment-2023-300843)

Appendix 1

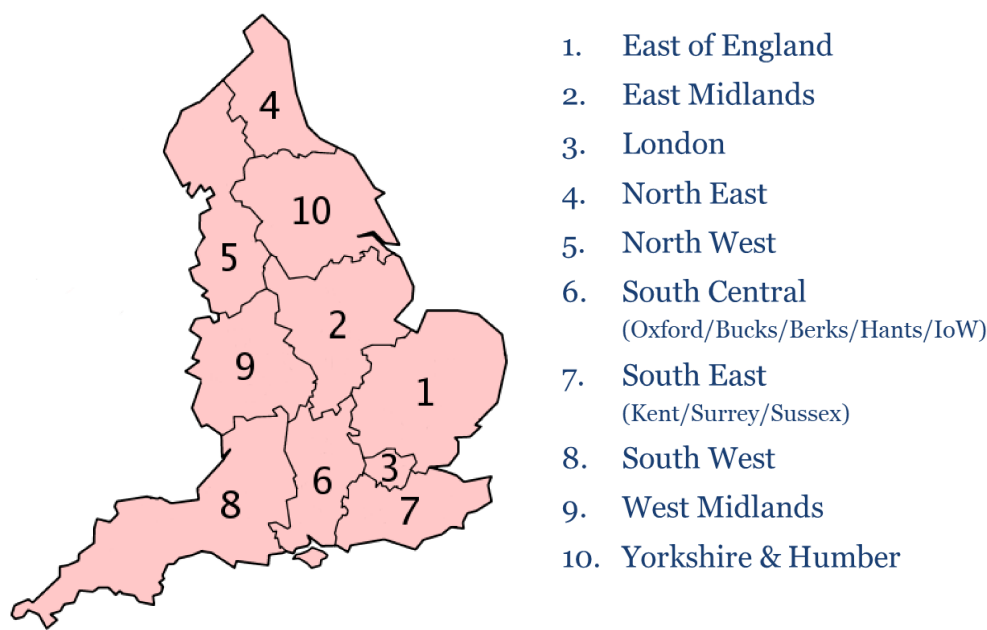

Figure 4 A map of the 10 NHS regions in England

Supplement: Supplementary data [file bmjment-2023-300843supp001.pdf]
